# Effects of Olanzapine on Bone Mineral Density, Glucose, and Lipid Metabolism in Schizophrenia Patients

**DOI:** 10.1155/2019/1312804

**Published:** 2019-03-21

**Authors:** Mining Liang, Beibei Zhang, Lu Deng, Rong Xu, Haishan Wu, Jindong Chen

**Affiliations:** ^1^Clinical Nursing Teaching and Research Section, The Second Xiangya Hospital, Central South University, Changsha, Hunan Province, China; ^2^Department of Psychiatry, The Second Xiangya Hospital, Central South University, Changsha, Hunan 410011, China; ^3^Mental Health Institute of the Second Xiangya Hospital, Central South University, Changsha, Hunan 410011, China; ^4^Chinese National Clinical Research Center for Mental Disorder (Xiangya), Chinese National Technology Institute on Mental Disorders, Hunan Key Laboratory of Psychiatry and Mental Health, Changsha, Hunan 410011, China; ^5^Brain Hospital of Hunan Province, Changsha, Hunan Province, China; ^6^Institute of Metabolism and Endocrinology, The Second Xiangya Hospital, Central South University, Changsha, China; ^7^Department of Neurosurgery, Xiangya Hospital, Central South University, Changsha, China

## Abstract

**Aim:**

To explore whether olanzapine alters bone mineral density (BMD), glucose, and lipid metabolism in schizophrenia patients.

**Methods:**

This study enrolled 150 patients diagnosed with schizophrenia according to *Diagnostic and Statistical Manual of Mental Disorders (DSM-IV)*, including 101 patients who had over 6-month history of olanzapine use (olanzapine-treated group) and 49 patients who had no history of antipsychotic use (first episode drug-naïve group). 71 subjects with age- and gender-matched healthy volunteers (healthy control group) were also enrolled. All study subjects were from the Chinese Han population recruited in the Second Xiangya Hospital from January 2015 to January 2016. Demographic and physical examination data were collected from all subjects. BMD measurements of the radius+ulna, lumbar spine (L1-4), and left hip were performed via a dual-energy X-ray absorptiometry test. Serum lipid, glucose, and insulin levels were analyzed. Psychopathology profiles in all enrolled schizophrenia patients were assessed by the positive and negative syndrome scale (PANSS).

**Results:**

There was no significant difference in age, gender, activity intensity, smoking, or drinking among the three groups. In the majority of evaluated bone areas, the BMD values in olanzapine-treated or drug-naïve patients were lower than those in the control group. However, BMD values in the drug-naïve group showed no difference or even decreased as compared with those in the olanzapine-treated group. Among the olanzapine-treated group, although not observed in every tested region, a positive correlation was found of BMI or HOMA-IR with BMD. Stepwise multiple linear regression analysis revealed independent predictive factors associated with BMD in groups/subgroups of schizophrenia patients or healthy controls, including gender, TG, BMI, body weight, HOMA-IR, and FBG.

**Conclusions:**

Schizophrenia, but not the long-term use of olanzapine, correlates with BMD loss in schizophrenia patients. Elevated BMI, TG, FBG, and insulin levels might protect these patients against bone degradation. Our work provides new information to improve the understanding, prevention, and treatment of osteoporosis in schizophrenia patients.

## 1. Introduction

Osteoporosis is a progressive metabolic bone disease characterized by the decrease of bone mineral density (BMD) and degradation of bone tissue. Osteoporosis is linked to the risk of bone fracture, and its incidence is rising worldwide [1]. Many factors contribute to the formation of osteoporosis, including genetically predetermined peak bone mineral density, age, gender, delayed menarche, premature menopause, insufficient calcium intake, lack of exercise, excessive smoking, excessive drinking, and caffeine abuse [2]. The proper management of osteoporosis risk factors could greatly reduce osteoporosis-associated mortality and morbidity rates.

Schizophrenia is a severe and chronic relapsing mental disorder with marked functional impairments. Due to the etiology and treatment of schizophrenia, the incidence of many health problems, such as cardiovascular diseases, metabolic syndrome, obesity, sexual disorders, and osteoporosis, is much higher in schizophrenia patients than in the general population [3]. Schizophrenia patients are prone to osteoporosis because of inadequate physical activity, abnormal eating, smoking and drinking habits, and insufficient sunlight exposure [4]. A 2012 review demonstrates that, in the majority of studies analyzed, reduced BMD or increased osteoporosis incidence is found in at least one bone area or one subgroup of schizophrenia patients treated with antipsychotics, as compared with healthy controls or population-based samples [5]. In addition, the recent studies have indicated that the use of antipsychotics might be associated with decreased BMD [6]. Increase in bone fracture risk has been specifically linked to the use of hyperprolactinemia-inducing antipsychotics [7], possibly due to altered bone turnover [8] and changed bone metabolism caused by an elevated prolactin level [9].

In clinical practice, atypical antipsychotics, such as olanzapine and risperidone, are widely used due to their less extrapyramidal side effects and better therapeutic efficiency as compared with typical antipsychotics [10]. However, body weight increase, glucose, and lipid metabolism disorders associated with the usage of atypical antipsychotics greatly decrease patients' compliance. A recent meta-analysis shows that olanzapine has a dramatic effect in upregulating body weight, glucose, and triglyceride (TG) in schizophrenia patients [11]. Importantly, the body weight or body mass index (BMI) inducing effect of atypical antipsychotics could counteract their BMD-reducing effect in schizophrenia patients [12]. Therefore, to better understand the overall impact of atypical antipsychotics in schizophrenia patients, it is essential to systematically analyze the interactions between bone metabolism dysregulation and glucose and lipid metabolic disorders associated with atypical antipsychotics.

The aim of this study was to compare BMD and indexes of glucose and lipid metabolism among long-term olanzapine-treated schizophrenia patients, first episode drug-naïve schizophrenia patients, and healthy controls among Chinese population. We expect our work to provide important information of the overall role of olanzapine in regulating bone mineral density, glucose, and lipid metabolism in schizophrenia patients.

## 2. Materials and Methods

### 2.1. Subjects and Groups

This study included 150 patients, enrolled from the Second Xiangya Hospital, Central South University, aged 18 to 40 years old, and diagnosed with schizophrenia in accordance with the diagnostic criteria outlined in the *Diagnostic and Statistical Manual of Mental Disorders IV (DSM-IV)* (American Psychiatric Association) [13]. 71 age- and gender-matched healthy control subjects with no major psychopathology (control group, *n* = 71) were also recruited. Patients with schizophrenia were further divided into olanzapine-treated subjects (olanzapine-treated group, *n* = 101), who had >5 years of disease duration and were treated with olanzapine for at least 6 months, and first episode drug-naïve subjects (drug-naïve group, *n* = 49), who had <2 years of disease duration and no history of antipsychotic usage. Exclusion criteria for all three groups included the following: severe cardiovascular, hepatic, or renal diseases that affect bone metabolism, such as diabetes and hyperthyroidism; diagnosis with a psychotic disease other than schizophrenia; usage of medications that might cause osteoporosis, such as mood stabilizers, antidepressant, or antianxiety drugs within 3 months prior to the enrollment; abnormal sex hormone levels (testosterone < 10 nmol/L in male subjects or <6 menstrual cycles per year in female subjects); and history of bone fracture within one year prior to the enrollment. This study was approved by the ethic committee of the Second Xiangya Hospital, Central South University. Written informed consent for participation in the study was obtained from all subjects after the study procedure had been fully explained.

The olanzapine-treated group (*n* = 101) was later divided based on either BMI value, including normal (18.5 < BMI < 24, *n* = 50), overweight (24 ≤ BMI < 28, *n* = 33), and obesity (BMI ≥ 28, *n* = 18) subgroups [14], or on HOMA-IR (homeostasis model assessment to evaluate insulin resistance) values, including IR (HOMA-IR > 2.68, *n* = 58) and non-IR (HOMA-IR ≤ 2.68, *n* = 43) subgroups. HOMA‐IR = [fasting insulin (*μ*U/mL) × fasting glucose (mmol/L)]/22.5 [15].

### 2.2. General Patients' Information

Detailed retrospective interviews were administered by psychiatrists to participants who met the study criteria to obtain information on smoking and drinking history, physical activity, family history of bone fractures, height, weight, age, diet, marital status, duration of illness, duration of antipsychotic use, and antipsychotic dosage. Physical activity was evaluated by physical activity index (intensity × duration × times). Positive and negative syndrome scale (PANSS) [16] was used to rate clinical symptoms in the 150 schizophrenia patients enrolled.

### 2.3. Bone Mineral Density Measurement

According to World Health Organization guidelines, BMD values at the femoral neck, lumbar spine, and total hip regions are primarily used as the reference standard of osteoporosis [17]. In the current study, BMD was measured with the Discovery WI dual-energy X-ray absorptiometry (DEXA) (Hologic, USA) in a total of 13 bone regions, including the lumbar (L1, L2, L3, and L4), total lumbar region (L1-4), left femoral neck (NECK), left trochanter (TROCH), Ward's triangle of the proximal left femur (WARD), total hip area (T-HIP), and the one-third distal (1/3), ultradistal (UD), middle distal (MID), and total region (TOTAL) of the right radius+ulna.

### 2.4. Biochemical Assessment

Fasting venous blood samples for all subjects (5 mL from each participant) enrolled in this study were drawn between 7 : 00 and 9 : 00 AM, placed in anticoagulant tubes, and left at room temperature for 20 min. Serum was separated by centrifugation for 5 min at 3500 rpm/min and analyzed in a clinical biochemical laboratory. Serum levels of total cholesterol (CHOL), triglyceride (TG), high-density lipoprotein (HDL), low-density lipoprotein (LDL), and fasting blood glucose (FBG) were measured by the commercially available immunoassay kits purchased from MeiKang Medical System (Ningbo, China) and a Hitachi Biochemical Analyzer 7170A (Japan). The insulin level was determined by the chemiluminescence immunoassay method using a Cobase 601 analyzer (Roche, Germany).

### 2.5. Statistical Analyses

Statistical analyses were performed using the SPSS software version 17.0 (SPSS, Chicago, IL). ANOVA analysis was used to compare variables of general patients' information and biochemical or BMD data among three different groups (olanzapine *vs.* drug-naïve *vs.* control; normal weight *vs.* overweight *vs.* obesity). The Student *t*-test was used to compare variables of general patients' information and biochemical or BMD data between two different groups (IR *vs.* non-IR). The chi-square test was used to compare categorical variables in different groups. All data were shown as mean ± SD, and a *p* value of <0.05 was considered statistically significant.

Stepwise multiple linear regression analyses were performed with BMD as a dependent variable to identify independent determinants for BMD. The results of all multiple linear regression analyses were expressed as *β* coefficients.

## 3. Results

### 3.1. Characterization of Sociodemographic Features

Table 1 shows the demographic data for all participants (*n* = 221). There were no significant differences between the three groups in age, gender, physical activity, and smoking or drinking habits. However, there were significant differences between the groups in body weight and BMI. The BMI or body weight of the olanzapine-treated group was significantly higher than that of the drug-naïve or control group (*p* < 0.001). A statistically significant longer duration of illness and a lower PANSS value (indicating better management of schizophrenia) were found in the olanzapine-treated group, as compared with the drug-naïve group.

### 3.2. Assessment of Indexes of Glucose and Lipid Metabolism

The glucose and lipid metabolic parameters for all participants (*n* = 221) are shown in Table 2. The TG, CHOL, LDL, FBG, and insulin levels of the olanzapine-treated group were significantly higher than those of the drug-naïve or control group (*p* < 0.001). The TG and LDL levels of the drug-naïve group were significantly higher than those of the control group (*p* < 0.001). The HDL level in the olanzapine or drug-naïve group was higher than that in the control group (*p* < 0.001). There were no significant differences in CHOL, FBG, or insulin levels between drug-naïve and control groups.

### 3.3. Assessment of Bone Mineral Density

Table 2 also shows the BMD values in the right radius+ulna, lumbar, and left femoral regions as measured for the three groups. A statistically significant decrease in BMD was found in the olanzapine or drug-naïve group as compared to the control group in 6 out of 13 evaluated bone regions, including MID, UD, TOTAL, L3, NECK, and TROCH (*p* < 0.05). No difference was found between the olanzapine and drug-naïve groups in the above 6 areas. BMD values in 3 out of 13 evaluated bone regions, including L1, L2, and T-HIP, were not different among the three groups. A significant decrease in the BMD values of L4 and L1-4 (total lumbar) was seen in the drug-naïve group as compared to that in the olanzapine or control group.

### 3.4. Subgroup Analysis Based on BMI

We further divided the olanzapine-treated group (*n* = 101) into normal weight (*n* = 50), overweight (*n* = 33), and obesity (*n* = 18) subgroups. Except for body weight and BMI that were significantly different among the three subgroups, all other sociodemographic and clinical characteristics analyzed were comparable between the subgroups (Table 3).

In addition, the TG, FBG, and insulin levels in the obesity subgroup were significantly higher than those in the normal weight subgroup (Table 4), suggesting a positive correlation between body weight/BMI and TG, FBG, or insulin in olanzapine-treated schizophrenia patients. BMD values were significantly increased in the obesity subgroup as compared to the normal weight subgroup in 7 out of 13 evaluated bone regions, including L1, L2, L3, NECK, TROCH, WARD, and T-HIP (Table 4).

### 3.5. Subgroup Analysis Based on HOMA-IR

Olanzapine-treated group was also divided into IR (*n* = 58) and non-IR (*n* = 43) subgroups. Comparison of the sociodemographic and clinical characteristics between the subgroups was shown in Table 5. A significant higher body weight and BMI were found in the IR subgroup as compared to the non-IR subgroup (Table 5). Significantly higher dosage of olanzapine use and longer duration of illness were seen in the IR subgroup as compared to the non-IR subgroup.

Moreover, the TG, CHOL, FBG, and insulin levels in the IR subgroup were significantly higher than those in the non-IR subgroup (Table 6). BMD values were significantly increased in the IR subgroup as compared to the non-IR subgroup in 5 out of 13 evaluated bone regions, including L1, NECK, TROCH, WARD, and T-HIP (Table 6).

### 3.6. Factors Affecting BMD in the Study Population

In all schizophrenia patients (*n* = 150), stepwise multiple linear regression analysis results showed factors that may affect BMD (Table 7). A negative correlation was found between the female gender and BMD in 1/3, MID, or TOTAL regions of the right radius+ulna. Compared to male patients, female patients might be more prone to BMD loss in the radius+ulna region. A positive correlation was found between body weight/BMI and BMD in 9 out of 13 evaluated bone regions, including 1/3, MID, TOTAL, L1, L2, NECK, TROCH, WARD, and T-HIP. A positive correlation was found between HOMA-IR and BMD in 6 out of 13 evaluated bone regions, including L1, L3, NECK, TROCH, WARD, and T-HIP.

In IR olanzapine-treated schizophrenia patients (*n* = 58) (Table 8), a positive correlation was found between body weight and BMD in 4 out of 13 evaluated bone regions, including 1/3, MID, TOTAL, and L1-4. A positive correlation was found between TG and BMD in 3 out of 13 evaluated bone regions, including 1/3, MID, and TOTAL. A negative correlation was found between HDL and BMD in L1-4.

In the first episode drug-naïve schizophrenia patients (*n* = 49), a positive correlation between TG or body weight and BMD was found in all 4 evaluated radius+ulna bone regions (Table 9).

In the healthy controls (*n* = 71), a positive correlation between BMI and BMD was found in the L2 region (Table 10). Interestingly, a negative correlation between LDL and BMD was found in the L2 and L3 regions.

## 4. Discussion

Our literature search revealed little information correlating antipsychotic-related glucose and lipid metabolic dysfunction and bone metabolism dysregulation in schizophrenia patients. In our study, schizophrenia patients with long-term treatment of olanzapine were compared with the first episode drug-naïve schizophrenia patients or healthy controls of the same age and gender in terms of lifestyle risk factors known to effect osteoporosis (smoking, drinking, exercise, body weight, and BMI), indexes of glucose and lipid metabolism (TG, CHOL, HDL, LDL, FBG, and insulin), and BMD in a total of 13 representing bone regions. We found no differences in age, gender, physical activity, and smoking or drinking habits among the three groups. Body weight/BMI was significantly higher in the olanzapine-treated group when compared to the other two groups and was similar between the other two antipsychotic-free groups, confirming that olanzapine treatment alone causes body weight gain. Olanzapine-induced weight gain has been attributed to increased food intake and metabolic dysfunctions [18]. Two major signaling pathways mediating olanzapine-induced weight gain have been proposed. Kim et al. [19] showed that olanzapine and clozapine selectively activated hypothalamic AMPK, a positive regulator of food intake [20], via a histamine H1 receptor. On the other hand, Lord's group [21] reported in an experimental female C57BL/6 mouse model that olanzapine-induced weight gain was mediated by the serotonin 2C receptor (HTR2C) and that the HTR2C agonist could inhibit olanzapine-induced hyperphagia and weight gain. However, the detailed mechanism underlying metabolic dysfunction-dependent weight gain induced by olanzapine remains unclear.

One of the major findings of this study is that in the majority of evaluated bone regions (8/13 regions), the BMD values in olanzapine and drug-naïve patients are lower than those in healthy controls (Table 2), demonstrating the presence of BMD loss in schizophrenia patients as shown by many other studies [5, 7, 22]. However, BMD values in the drug-naïve group were similar (11/13 regions) or even higher (2/13 regions) than those in the olanzapine-treated group (Table 2). These findings are consistent with the previous study demonstrating clinically relevant decrease of BMD in response to risperidone, but not olanzapine treatment. Such effect is presumably associated with the hyperprolactinemic effect of these atypical antipsychotics, which is high in risperidone and low in olanzapine [23]. Indeed, studies have shown that prolactin-elevating antipsychotics, such as risperidone, penetrate the blood-brain barrier poorly, therefore occupying the D2 receptors much longer in the pituitary (peripheral) than the striatum (central) and leading to a prolonged hyperprolactinemia, which is closely correlated with BMD loss. On the other hand, prolactin-sparing antipsychotics, such as olanzapine, do not have long-lasting D2 antagonism effects and do not cause long-term hyperprolactinemia [24, 25], therefore leading to minimal loss of BMD.

In order to correlate olanzapine-induced glucose and lipid metabolic dysfunction and bone metabolism dysregulation in schizophrenia patients, we further divided olanzapine-treated schizophrenia patients into subgroups based on their BMI (relation to obesity) or HOMA-IR (relation to insulin resistance) values. We found that obesity was positively correlated with glucose dysmetabolism and dyslipidemia in olanzapine-treated schizophrenia patients but was linked to increased BMD in the majority of lumbar and left femoral bone areas of these patients. Interestingly, insulin resistance was positively related to glucose dysmetabolism and dyslipidemia in olanzapine-treated schizophrenia patients but was also associated with elevated BMD in all left femoral bone areas examined in these patients. The positive correlations between obesity or insulin resistance and BMD were consistent with the traditional viewpoint that fat mass protects bone health [26, 27]. Similar to our results, the recent Tunisian study showed a higher BMD in obese women (compared to nonobese women) in the left and right femurs, total hip, and whole body and in insulin-resistant women (compared to non-insulin-resistant women) in the left femur and total hip [28]. The following hypothetical mechanisms are thought to underline the relationship between obesity and BMD. Steppan's group showed in the ob/ob mice model that obesity-induced bone growth was mediated by leptin, the product of the obese gene, via its signaling receptor (ObRb) [29]. Lamghari et al. showed that the leptin hormone plays an important role on bone mass through modulating positively OPG/RANKL balance [30]. Meanwhile, Yamauchi et al. found that there was a significant and positive relationship between the plasma leptin levels and BMD values at all skeleton sites measured among postmenopausal women, suggesting that leptin could associate in maintaining bone mass and bone quality [31]. However, the recent evidence suggests that obesity increases risk of bone fracture in a BMD-independent manner [32]. Therefore, better techniques of the proper assessment of bone strength/quality and a more accurate interpretation of obesity and insulin resistance indexes, as well as a better understanding of the interconnection network (e.g., hormones and cytokines) between adipose and bone tissues are of great importance in elucidating the relationship between obesity/insulin resistance and bone health.

Factors that may affect BMD were explored using stepwise multiple linear regression analyses in different groups or subgroups, and several interesting points were found. The female gender is negatively correlated with BMD in the majority of the right radius+ulna bone areas of schizophrenia patients, suggesting a higher susceptibility of BMD loss in the radius+ulna region in female schizophrenia patients as compared to male patients. Body weight/BMI is positively correlated with BMD in schizophrenia patients, drug-naïve schizophrenia patients, and healthy controls, although affecting different bone areas. HOMA-IR is an independent positive variable affecting BMD in schizophrenia patients, but not in drug-naïve schizophrenia patients or healthy controls. These data further confirm a complex network among glucose and lipid metabolic dysfunction, bone metabolism dysregulation, and the pathology of schizophrenia.

The limitations of our work include a lack of information on other factors that may also contribute to osteoporosis, such as nutrition, calcium/vitamin D intake, and levels of parathyroid hormone and prolactin [33]. Also, our sample size is relatively small. We would expand our future work to incorporate larger sample size, a more comprehensive list of osteoporosis-related factors and pathological conditions. In conclusion, we found that schizophrenia disease, but not long-term usage of olanzapine, correlates closely with BMD loss and osteoporosis in Chinese schizophrenia patients. Body weight and insulin resistance protect these schizophrenia patients against bone degradation. Female schizophrenia patients are more prone to BMD loss in the radius+ulna region. Altogether, our work reveals new information in the interaction between atypical antipsychotic-induced glucose and lipid metabolic dysfunction and bone metabolism dysregulation in schizophrenia patients, which could improve our understanding in the prevention and treatment of osteoporosis in schizophrenia patients.

## Figures and Tables

**Table 1:** Sociodemographic and clinical characteristics of the study population.

	Olanzapine-treated group (*N* = 101)	First episode drug-naïve group (*N* = 49)	Healthy control (*N* = 71)	*p* ^#^	Multicomparison
Age (years)	26.1 ± 4.9	24.8 ± 4.9	25.9 ± 4.7	0.336	—
Gender (F/M)	55/46	28/21	40/31	0.974	—
Body weight (kg)	64.72 ± 10.02	57.00 ± 9.92	60.21 ± 7.82	<0.001	O > F, H
BMI	24.41 ± 3.73	21.84 ± 2.88	22.76 ± 1.67	<0.001	O > F, H
Physical activity index^a^	5.42 ± 3.16	5.82 ± 3.27	5.79 ± 4.38	0.893	—
Smoking (Y/N)	27/74	5/44	11/60	0.395	—
Drinking (Y/N)	17/84	6/43	3/68	0.232	—
Dosage (mg/d)^b^	452.8 ± 129.2	—	—	—	—
Duration of olanzapine use (year)	2.39 ± 1.05	—	—	—	—
Duration of illness (year)	4.9 ± 2.1	1.2 ± 0.6		<0.001	O > F
PANSS	91.9 ± 11.3	96.4 ± 10.5		0.021	O > F

^a^Physical activity index = intensity × duration × times. ^b^Olanzapine medication dosages were transformed to chlorpromazine equivalent dosages. ^#^Comparison of sociodemographic and clinical characteristics among the olanzapine-treated, first episode drug-naïve, and healthy control groups.

**Table 2:** Biochemical and BMD characteristics of the study population.

	Olanzapine-treated group (*N* = 101)	First episode drug-naïve group (*N* = 49)	Healthy control (*N* = 71)	*p* ^#^	Multicomparison
TG (mmol/L)	2.07 ± 1.63	1.18 ± 0.57	0.92 ± 0.42	<0.001	O > F > H
CHOL (mmol/L)	4.61 ± 0.85	4.16 ± 0.72	4.18 ± 0.66	<0.001	O > F, H
HDL (mmol/L)	2.06 ± 1.15	2.18 ± 0.94	1.41 ± 0.30	<0.001	O, F > H
LDL (mmol/L)	2.61 ± 0.72	1.91 ± 0.85	1.58 ± 0.64	<0.001	O > F > H
FBG (mmol/L)	5.30 ± 1.52	4.66 ± 0.61	4.60 ± 0.49	<0.001	O > F, H
Insulin (*μ*U/mL)	25.29 ± 23.26	11.37 ± 10.76	7.53 ± 3.75	<0.001	O > F, H
BMD, radius+ulna (g/cm^2^)					
1/3	0.68 ± 0.07	0.67 ± 0.08	0.72 ± 0.05	0.275	—
MID	0.56 ± 0.07	0.55 ± 0.07	0.64 ± 0.08	<0.001	O, F < H
UD	0.43 ± 0.13	0.41 ± 0.06	0.47 ± 0.03	<0.001	O, F < H
TOTAL	0.55 ± 0.07	0.54 ± 0.06	0.64 ± 0.09	<0.001	O, F < H
BMD, lumbar (g/cm^2^)					
L1	0.87 ± 0.09	0.84 ± 0.12	0.89 ± 0.08	0.023	F < H
L2	0.93 ± 0.11	0.90 ± 0.12	0.96 ± 0.08	0.018	F < H
L3	0.95 ± 0.12	0.93 ± 0.11	0.99 ± 0.08	0.005	O, F < H
L4	0.95 ± 0.12	0.91 ± 0.11	1.01 ± 0.08	<0.001	F < O < H
L1-4	0.94 ± 0.12	0.89 ± 0.12	0.97 ± 0.07	0.002	F < O, H
BMD, left hip (g/cm^2^)					
NECK	0.79 ± 0.11	0.76 ± 0.13	0.82 ± 0.07	0.003	O, F < H
TROCH	0.65 ± 0.09	0.62 ± 0.08	0.69 ± 0.07	<0.001	O, F < H
WARD	0.74 ± 0.13	0.73 ± 0.14	0.78 ± 0.08	0.066	—
T-HIP	0.88 ± 0.13	0.84 ± 0.11	0.91 ± 0.09	0.017	F < H

^#^Comparison of biochemical and BMD characteristics among the olanzapine-treated, first episode drug-naïve, and healthy control groups.

**Table 3:** BMI-based subgroup analysis of sociodemographic and clinical characteristics in the olanzapine-treated group (*n* = 101).

	Normal weight (*N* = 50)	Overweight (*N* = 33)	Obesity (*N* = 18)	*p* ^∗^	Multicomparison
Age (years)	26.2 ± 6.9	28.1 ± 5.2	28.5 ± 6.2	0.303	—
Gender (F/M)	29/21	19/15	8/6	0.982	—
Body weight (kg)	58.62 ± 7.14	68.29 ± 5.95	78.92 ± 12.14	<0.001	No < Ov < Ob
BMI	21.86 ± 1.28	25.74 ± 1.18	30.56 ± 2.02	<0.001	No < Ov < Ob
Physical activity index^a^	5.10 ± 4.52	4.45 ± 4.04	5.64 ± 4.27	0.887	—
Smoking (Y/N)	15/35	8/25	4/10	0.847	
Drinking (Y/N)	11/39	5/28	1/13	0.393	—
Dosage (mg/d)^b^	422.5 ± 137.6	384.9 ± 129.2	485.71 ± 183.3	0.087	—
Duration of olanzapine use (year)	2.7 ± 2.1	2.4 ± 1.9	2.5 ± 2.4	0.892	—
Duration of illness (year)	5.6 ± 4.7	5.8 ± 4.9	4.1 ± 3.9	0.585	—
PANSS	90.2 ± 10.3	89.4 ± 11.2	92.6 ± 13.7	0.429	—

^a^Physical activity index = intensity × duration × times. ^b^Olanzapine medication dosages were transformed to chlorpromazine equivalent dosages. ^∗^Comparison of sociodemographic and clinical characteristics among the normal weight, overweight, and obesity subgroups.

**Table 4:** BMI-based subgroup analysis of biochemical and BMD characteristics in the olanzapine-treated group (*n* = 101).

	Normal weight (*N* = 50)	Overweight (*N* = 33)	Obesity (*N* = 18)	*p* ^∗^	Multicomparison
TG (mmol/L)	1.55 ± 1.06	2.18 ± 1.82	2.66 ± 2.07	0.030	No < Ob
CHOL (mmol/L)	4.34 ± 0.82	4.43 ± 0.99	4.79 ± 0.68	0.241	—
HDL (mmol/L)	1.86 ± 1.05	1.68 ± 1.06	2.33 ± 1.24	0.162	—
LDL (mmol/L)	1.93 ± 0.72	2.01 ± 0.93	1.87 ± 0.90	0.851	—
FBG (mmol/L)	4.90 ± 0.81	5.25 ± 0.83	6.47 ± 2.94	0.001	No, Ov < Ob
Insulin (*μ*U/mL)	17.89 ± 10.56	28.75 ± 20.91	22.41 ± 18.76	0.041	No < Ob
BMD, radius+ulna (g/cm^2^)					
1/3	0.67 ± 0.08	0.69 ± 0.07	0.68 ± 0.05	0.718	—
MID	0.55 ± 0.08	0.57 ± 0.07	0.67 ± 0.06	0.518	—
UD	0.43 ± 0.14	0.39 ± 0.10	0.43 ± 0.04	0.373	—
TOTAL	0.55 ± 0.07	0.56 ± 0.06	0.56 ± 0.07	0.583	—
BMD, lumbar (g/cm^2^)					
L1	0.84 ± 0.09	0.88 ± 0.08	0.94 ± 0.07	<0.001	No, Ov < Ob
L2	0.89 ± 0.12	0.95 ± 0.11	1.01 ± 0.06	0.001	No, Ov < Ob
L3	0.91 ± 0.11	0.96 ± 0.11	1.01 ± 0.09	0.009	No < Ob
L4	1.14 ± 1.62	1.34 ± 1.12	1.03 ± 0.09	0.818	—
L1-4	1.14 ± 0.78	1.36 ± 1.12	0.99 ± 0.08	0.808	—
BMD, left hip (g/cm^2^)					
NECK	0.74 ± 0.09	0.79 ± 0.12	0.85 ± 0.08	0.004	No, Ov < Ob
TROCH	0.62 ± 0.09	0.65 ± 0.11	0.72 ± 0.05	0.003	No, Ov < Ob
WARD	0.71 ± 0.11	0.75 ± 0.15	0.82 ± 0.08	0.019	No < Ob
T-HIP	0.83 ± 0.13	0.91 ± 0.11	0.98 ± 0.08	<0.001	No < Ov < Ob

^∗^Comparison of biochemical and BMD characteristics among the normal weight, overweight, and obesity subgroups.

**Table 5:** HOMA-IR-based subgroup analysis of sociodemographic and clinical characteristics in the olanzapine-treated group (*n* = 101).

	IR (*n* = 58)	Non-IR (*n* = 43)	*p*
Age (years)	26.5 ± 5.7	27.8 ± 7.1	0.291
Gender (F/M)	31/27	24/19	0.813
Body weight (kg)	66.44 ± 11.4	61.38 ± 9.01	0.018
BMI	25.18 ± 3.81	22.80 ± 2.71	0.001
Physical activity index^a^	5.12 ± 2.91	5.26 ± 3.76	0.936
Smoking (Y/N)	17/41	10/33	0.497
Drinking (Y/N)	10/48	7/36	0.559
Dosage (mg/d)^b^	463.4 ± 127.9	389.7 ± 145.1	0.009
Duration of olanzapine use (year)	1.82 ± 1.06	2.69 ± 1.67	0.068
Duration of illness (year)	6.55 ± 4.19	3.55 ± 2.36	0.002
PANSS	92.6 ± 12.5	93.2 ± 11.8	0.468

^a^Physical activity index = intensity × duration × times. ^b^Olanzapine medication dosages were transformed to chlorpromazine equivalent dosages.

**Table 6:** HOMA-IR-based subgroup analysis of biochemical and BMD characteristics in the olanzapine-treated group (*n* = 101).

	IR (*n* = 58)	Non-IR (*n* = 43)	*p*
TG (mmol/L)	2.32 ± 1.81	1.39 ± 0.88	0.001
CHOL (mmol/L)	4.64 ± 0.75	4.19 ± 0.96	0.011
HDL (mmol/L)	2.01 ± 1.15	1.67 ± 1.04	0.125
LDL (mmol/L)	1.91 ± 0.82	1.98 ± 0.81	0.688
FBG (mmol/L)	5.66 ± 1.64	4.63 ± 0.63	<0.001
Insulin (*μ*U/mL)	32.01 ± 24.07	7.87 ± 3.14	<0.001
BMD, radius+ulna (g/cm^2^)			
1/3	0.67 ± 0.06	0.67 ± 0.08	0.698
MID	0.57 ± 0.06	0.56 ± 0.08	0.787
UD	0.43 ± 0.08	0.41 ± 0.15	0.462
TOTAL	0.55 ± 0.06	0.55 ± 0.08	0.964
BMD, lumbar (g/cm^2^)			
L1	0.88 ± 0.09	0.84 ± 0.09	0.044
L2	0.94 ± 0.11	0.90 ± 0.12	0.144
L3	0.96 ± 0.11	0.91 ± 0.11	0.059
L4	1.37 ± 2.23	0.91 ± 0.12	0.180
L1-4	1.39 ± 2.44	0.89 ± 0.11	0.182
BMD, left hip (g/cm^2^)			
NECK	0.81 ± 0.11	0.73 ± 0.08	<0.001
TROCH	0.67 ± 0.10	0.61 ± 0.07	0.005
WARD	0.77 ± 0.13	0.69 ± 0.11	0.002
T-HIP	0.91 ± 0.11	0.82 ± 0.14	0.001

**(a):** 

BMD, radius+ulna	1/3		MID		TOTAL	
	*β*	*p*	*β*	*p*	*β*	*p*
Gender (Female)	−0.072	<0.001	−0.059	0.001	−0.062	<0.001
Body weight	0.002	0.005	0.002	0.002	0.002	0.002
TG	0.011	0.014	0.011	0.018	0.010	0.028

BMD, lumbar	L1		L2		L3	
	*β*	*p*	*β*	*p*	*β*	*p*
HOMA-IR	0.003	0.046	—	—	0.004	0.031
Body weight	0.004	<0.001	—	—	—	—
BMI	—	—	0.013	0.001	—	—

**(b):** 

BMD, left hip	NECK		TROCH	
	*β*	*p*	*β*	*p*
Body weight	0.004	0.001	0.003	0.001
HOMA-IR	0.005	0.003	0.054	0.006
FBG	—	—	0.019	0.007

BMD, left hip	T-HIP		WARD	
	*β*	*p*	*β*	*p*

Body weight	0.005	<0.001	0.003	0.011
HOMA-IR	0.006	0.002	0.004	0.018

**(a):** 

BMD, radius+ulna	1/3		MID		TOTAL	
	*β*	*p*	*β*	*p*	*β*	*p*
TG	0.012	0.006	0.011	0.014	0.001	0.013
Body weight	0.001	0.042	0.002	0.024	0.002	0.015

**(b):** 

BMD, lumbar	L1		L1-4	
	*β*	*p*	*β*	*p*
Body weight	0.003	0.002	0.004	0.001
HDL	—	—	−0.039	0.002
FBG			0.023	0.007

**Table 9:** Stepwise multiple linear regression analyses of variables affecting BMD in first episode drug-naïve schizophrenia patients (*n* = 49).

BMD, radius+ulna	1/3		MID	
	*β*	*p*	*β*	*p*
TG	0.041	0.041	0.038	0.031
Body weight (kg)	0.003	0.014	0.002	0.048

BMD, radius+ulna	UD		TOTAL	
	*β*	*p*	*β*	*p*
TG	0.031	0.042	0.037	0.032
Body weight (kg)	0.002	0.049	0.002	0.030

**Table 10:** Stepwise multiple linear regression analyses of variables affecting BMD in healthy controls (*n* = 71).

BMD, lumbar	L2		L3	
	*β*	*p*	*β*	*p*
LDL	−0.030	0.034	−0.033	0.012
BMI	0.017	0.006		

## Data Availability

The data used to support the findings of this study are included within the article.
